# Super-Resolution of Dental Panoramic Radiographs Using Deep Learning: A Pilot Study

**DOI:** 10.3390/diagnostics13050996

**Published:** 2023-03-06

**Authors:** Hossein Mohammad-Rahimi, Shankeeth Vinayahalingam, Erfan Mahmoudinia, Parisa Soltani, Stefaan J. Bergé, Joachim Krois, Falk Schwendicke

**Affiliations:** 1 Topic Group Dental Diagnostics and Digital Dentistry, ITU/WHO Focus Group AI on Health, 10117 Berlin, Germany; 2 Department of Oral and Maxillofacial Surgery, Radboud University Nijmegen Medical Centre, 6525 GA Nijmegen, The Netherlands; 3 Department of Computer Engineering, Sharif University of Technology, Tehran 11155, Iran; 4 Department of Oral and Maxillofacial Radiology, Dental Implants Research Center, Dental Research Institute, School of Dentistry, Isfahan University of Medical Sciences, Isfahan 81746, Iran; 5 Department of Oral Diagnostics, Digital Health and Health Services Research, Charité—Universitätsmedizin Berlin, 10117 Berlin, Germany

**Keywords:** super-resolution, neural networks, deep learning, image enhancement, panoramic radiographs

## Abstract

Using super-resolution (SR) algorithms, an image with a low resolution can be converted into a high-quality image. Our objective was to compare deep learning-based SR models to a conventional approach for improving the resolution of dental panoramic radiographs. A total of 888 dental panoramic radiographs were obtained. Our study involved five state-of-the-art deep learning-based SR approaches, including SR convolutional neural networks (SRCNN), SR generative adversarial network (SRGAN), U-Net, Swin for image restoration (SwinIr), and local texture estimator (LTE). Their results were compared with one another and with conventional bicubic interpolation. The performance of each model was evaluated using the metrics of mean squared error (MSE), peak signal-to-noise ratio (PNSR), structural similarity index (SSIM), and mean opinion score by four experts (MOS). Among all the models evaluated, the LTE model presented the highest performance, with MSE, SSIM, PSNR, and MOS results of 7.42 ± 0.44, 39.74 ± 0.17, 0.919 ± 0.003, and 3.59 ± 0.54, respectively. Additionally, compared with low-resolution images, the output of all the used approaches showed significant improvements in MOS evaluation. A significant enhancement in the quality of panoramic radiographs can be achieved by SR. The LTE model outperformed the other models.

## 1. Introduction

Panoramic radiography is one of the most common imaging techniques for dental purposes, with both maxillary and mandibular structures, including teeth, being visible [1,2]. One of the main issues that can significantly influence dental diagnosis and treatment planning is the resolution of panoramic radiographs, which varies among manufacturers and machine types and the regions of each image [3]. Since panoramic radiography combines scanning and tomography, the objects in the curved focal trough or image layer are typically visualized with the highest resolution. Image resolution gradually decreases as objects move further from the center (inward or outward) of the image layer. In general, the resolution of panoramic radiographs varies from 2.58 to 3.19 lp/mm horizontally and <1.88 to 3.19 lp/mm vertically in different panoramic machines and image areas [4]. Notably, clinically relevant objects are not always located in the ideal image layer due to patient positioning errors, anatomical constraints, and geometric properties. Hence, inadequate resolution remains a shortcoming in panoramic radiographs compared to intraoral projections, where resolutions >20 lp/mm can be achieved [5].

Most of the available commercial softwares for viewing and processing radiographic images are equipped with built-in zoom tools as an alternative for increasing the resolution. Zoom features generally work using interpolation techniques. Several studies have shown the applicability of zooming for diagnostic tasks, such as caries detection, linear measurements, and fracture detection [6,7,8]. However, an upper limit exists for magnification performance, above which the boundaries of anatomical structures and lesions are not detected correctly. Therefore, zooming only limitedly addresses the resolution limitations of panoramic radiographs.

Super-resolution (SR) is a classic problem in computer vision in which an image with a high resolution (HR) is recovered from an image with a low resolution (LR). Due to the growing popularity of deep learning, the number of SR approaches based on deep learning has increased significantly [9]. SR methods based on deep learning can enhance the radiographic resolution without the limitations of conventional zooming features while diagnostically acceptable LR images (with reduced complexity of image acquisition and lower radiation doses) [10].

In dentistry, a recent study successfully employed deep learning-based methods to achieve SR of periapical radiographs [11]. For panoramic radiographs, where SR seems even more warranted, evidence on deep learning for SR is not available. Consequently, we aimed to assess deep learning for the SR of dental panoramic radiographs and to compare it against a conventional approach to improving resolution.

## 2. Materials and Methods

### 2.1. Study Design

In the present study, five state-of-the-art deep learning-based SR approaches and one conventional SR approach were applied to dental panoramic radiographs to improve image resolution. Note that, in this pilot study, we did not focus on SR to support the detection of specific conditions, but on the generic (disease-agnostic) improvement of image assessability by deep learning-based SR.

Reporting follows the Checklists for Artificial Intelligence in Medical Imaging [12] and Artificial Intelligence in Dental Research [13]. For readers unfamiliar with deep learning and the associated terminology, a number of definitions employed in the present methods section are presented in Table 1.

### 2.2. Dataset and Data Preparation

In total, 888 dental panoramic radiographs were collected for this study from a private oral and maxillofacial radiology center in Tehran, Iran. A comprehensive sample from patients who visited the radiology center in June 2021 was used in this study, assuming that the number of images was sufficient to demonstrate the effects of SR in this exploratory study. Low-quality images (e.g., blurry or noisy images) were excluded (*n* = 31). All the images were taken using Planmeca ProMax (Planmeca, Helsinki, Finland). The device settings were 64–72 kVp, 6.3–12.5 mA, and 13.8–16 s exposure time. The images were exported to .jpg format with a size of 2943 × 1435. All the samples were anonymized before use in the study.

To assess whether deep learning and other conventional approaches were suitable for improving resolution, we downscaled all HR images by a factor of 4× to create LR images. Such generic downsampling has been previously employed to simulate LR images and allows high standardization and replicability [14]. The original HR images were considered as ground truth. Seventy percent of the images (*n* = 622) were selected as the training set. Of the remaining images, 50% were selected as a test set (*n* = 133), and the other 50% were selected as a validation set (*n* = 133).

### 2.3. Model Architectures

We applied five deep learning SR approaches, yielding SR images, and compared them against each other and conventional bicubic interpolation.

#### 2.3.1. Super-Resolution Convolutional Neural Networks (SRCNN)

Dong et al. [15] first introduced SCRNN in 2014. This network learns end-to-end LR-image-to-HR-image mapping using deep convolutional neural networks. The loss function of this model is mean squared error (MSE).

#### 2.3.2. Super-Resolution Generative Adversarial Network (SRGAN)

Ledig et al. [16] applied generative adversarial networks for SR tasks. In their approach, there is an adversarial loss and a content loss. A discriminator network is trained to distinguish between the SR and HR images through adversarial loss, pushing the solution into the HR image manifold. They also proposed a content loss based on perceptual rather than pixel similarity.

#### 2.3.3. U-Net

U-Net is a convolutional neural network (CNN) initially developed for medical image segmentation [17]. The main idea was to use a CNN (downsampling path) in conjunction with an upsampling component (upsampling path) to increase the resolution of the output image. The authors also proposed connecting opposing convolutional layers using skip connections. These connections would provide high-resolution features to the upsampling path. In this paper, we fed U-Net with LR images for the SR task without modifying its structure. However, we changed the loss function to MSE.

#### 2.3.4. Swin for Image Restoration (SwinIR)

SwinIR is a relatively new SR approach based on Swin transformers [18]. The SwinIR algorithm comprises three steps: shallow feature extraction, deep feature extraction, and high-quality image reconstruction. The deep feature extraction module consists of several residual Swin transformer blocks, each containing several layers of the Swin transformer with a residual connection.

#### 2.3.5. Local Texture Estimator (LTE)

LTE is the most recent approach to report favorable results with a shorter running time compared to current state-of-the-art models [19]. LTE is a dominant frequency estimator for natural images, which allows a continuous reconstruction of images with delicate details derived from an implicit function. It can accurately characterize image textures in 2D Fourier space when jointly trained with a deep SR architecture.

### 2.4. Training Details

The training was conducted on a Tesla K80 graphic processor unit (Nvidia Corporation, Santa Clara, CA, USA) using the Google Collaboratory platform. The Python programming language and PyTorch library were used for the model implementation.

After the initial assessments of each model, the number of epochs was set from 30 to 120 on the basis of the model’s performance on the validation set. To prevent overfitting, we used early stopping, where we saved the best model weights according to their performance on the validation dataset based on the structural similarity index (SSIM). Except for U-Net, all hyperparameters of the implemented approaches were set in their original implementation. Grid search was used for the hyperparameter tuning of U-Net for batch size, learning rate, and the optimizer [20].

### 2.5. Evaluation

Our models were run five times using different random seeds to reduce the possibility of randomness in the results. The mean and standard deviation of each metric are reported as a result [21,22]. For this study, four metrics were used to assess the performance of each SR approach, defined as follows [9]:

#### 2.5.1. Mean Squared Error (MSE)

MSE is defined as the mean of the squares of differences between the pixel values of HR and SR images. In other words, MSE calculates the difference between each pixel of the HR image and its corresponding pixel in the SR image. It is defined as follows:

(1) $$MSE=\frac{1}{MN}\overset{M}{\underset{i=1}{\sum }}\overset{N}{\underset{j=1}{\sum }}{\left(f-f^{\prime }\right)}^{2},$$

where *f* is the given HR image and *f′* is the reconstructed SR image of size *M* × *N*.

#### 2.5.2. Peak Signal-to-Noise Ratio (PSNR)

PSNR is calculated by dividing the highest value of an image by the power of distorting noise, here MSE, which determines the quality of the image representation. It is defined as follows:

(2) $$PSNR=10{log}_{10}\frac{{\left(255\right)}^{2}}{MSE},$$

#### 2.5.3. Structural Similarity Index (SSIM)

SSIM is concerned with the perception of quality by the human visual system. Here, three factors are considered: loss of correlation, luminance distortion, and contrast distortion. Each of them is calculated as follows:

(3) $$SSIM\left(f,f^{\prime }\right)=l\left(f,f^{\prime }\right).c\left(f,f^{\prime }\right).s\left(f,f^{\prime }\right),$$

where *f* is the given HR image and *f′* is the reconstructed SR image. Moreover, *I*, *c*, and *f* are loss of correlation coefficient, luminance distortion, and contrast distortion, respectively, which are calculated on the basis of a comparison of HR and SR images.

#### 2.5.4. Mean Objective Scale (MOS)

Unlike the other three metrics, MOS is a subjective evaluation of the model’s performance. Here, we asked four experienced dentists to independently score a random subset (15 images per model) of SR images selected from the test set. Dentists rated each image on a scale of 1 (bad quality) to 5 (optimal quality). To reduce bias, the images were presented randomly to the raters. The mean and standard deviation were reported. HR images were provided as control samples and rated similarly.

### 2.6. Statistical Analysis

The Python programming language and SciPy open-source scientific computing library [23] were used for statistical analysis. Using an unpaired two-tail *t*-test, we compared the models’ MSE, PSNR, and SSIM values. To compare the models’ outcome regarding MSE, PSNR, and SSIM values with the conventional bicubic approach, we used a one-sample *t*-test. Moreover, we used the Wilcoxon signed-rank test to evaluate differences in the MOS of the models compared to the conventional bicubic approach. Any *p*-values less than 0.05 were considered statistically significant. We also calculated the Pearson correlation coefficient (*R*-value) of the MSE, PSNR, and SSIM means against the MOS to assess which objective metric most closely reflects the subjective assessment of the clinicians. For interpretation, *R*-values of 0–0.10 are considered negligible, of 0.1–0.39 are considered weak, of 0.40–0.69 are considered moderate, of 0.70–0.89 are considered strong, and of 0.9–1 are considered very strong [24].

## 3. Results

**Figure 1:**
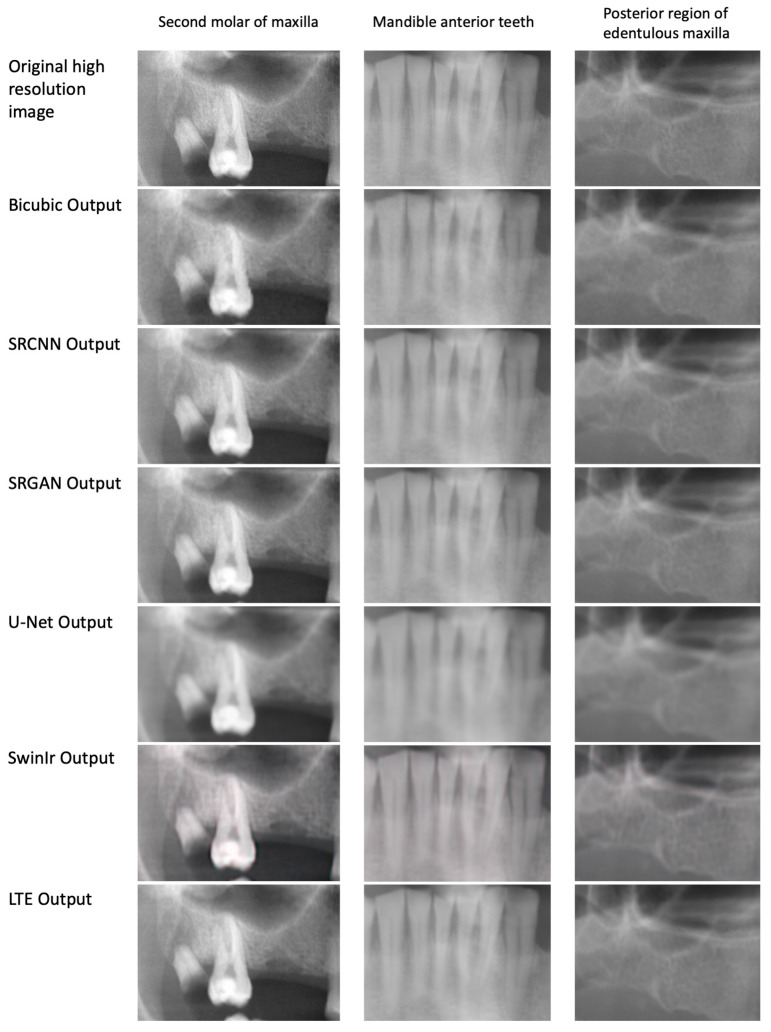
Super-resolution with different approaches.

The sample output of the trained models is presented in Figure 1. Table 2 provides an overview of the performance of different models on the test set. Moreover, Table 3 presents the results of the statistical analysis of the models’ comparison. Regarding the MSE metric, SRCNN and LTE showed better performances with MSEs of 7.48 ± 0.30 and 7.42 ± 0.44, respectively (*p* < 0.001). Similarly, these two models outperformed others when assessing the PSNR (39.57 ± 0.16 and 39.74 ± 0.17, respectively) (*p* < 0.001). For SSIM, all models were found to have similar performance (0.916–0.919) except for SRGAN, which showed a poorer outcome with SSIM of 0.901 ± 0.005 (*p* < 0.001). All deep learning models outperformed the bicubic baseline when assessing MSE, PSNR, and SSIM (with all *p* < 0.001, except for SRGAN in PSNR, which was 0.046).

The results of the MOS evaluation are presented in Table 4. MOS was significantly higher for all SR compared with conventionally restored images. In bicubic images (Figure 1), it was impossible to see the root canals of the second molar, while, in HR and most SR approaches (except U-Net and, to some degree, SwinIR), these were visible.

The *R*-values for MSE, PSNR, and SSIM against MOS were −0.794 (strong negative correlation), 0.773 (strong positive correlation), and 0.763 (strong positive correlation), respectively, i.e., all three metrics closely reflected the subjective assessments of the clinicians.

## 4. Discussion

In the present study, on a dataset of 888 dental panoramic radiographs, SRCNN, U-Net, and LTE performed better than other SR approaches when assessing MSE and PSNR. When considering SSIM as the metric, the difference between models was less clear. Subjective evaluation using MOS found that only SRGAN and LTE yielded significant resolution improvements.

Recently, SR approaches based on deep learning have been proposed to overcome the disadvantages of conventional interpolation-based methods for increasing image resolution. In the present study, we evaluated five deep learning-based SR approaches, some of which have been used before for medial image super-resolution [14]. Dong et al., proposed the SRCNN algorithm based on deep convolutional neural networks and reported its successful application in digital photographs [15]. Umehara et al., in a series of studies, successfully used SRCNN to increase the resolution of chest radiographs [25,26,27]. As discussed, Moran et al., employed SRCNN to enhance the quality of periapical radiographs [28]. Notably, for this purpose, they found that, while SRCNN improved the visual quality of radiographic images, its application was ineffective in enhancing the detection of periodontal bone loss in periapical radiographs. We confirmed this finding for SRCNN, which increased objective metrics, while clinicians did not find the resulting SR images to yield significantly better quality than LR. SRGAN is another SR model developed by Ledig et al. They reported that SRGAN could recover photo-realistic textures from down-sampled images, leading to significant gains in MOS values of image quality [16]. This is in line with our findings, where SRGAN improved MOS (something which has previously been found for periapical radiographs [11]), while objective metrics were not necessarily improved.

Other state-of-the-art SR models were evaluated. For instance, SwinIR, which uses Swin transformers as the backbone, has shown promising results for different SR tasks [18], especially for grayscale images (e.g., radiographs). Puttaguntaa et al. [29] reported that SwinIr outperformed SRGAN, BSRGAN, and RealESRGAN on chest radiographs. On the other hand, for reconstruction, U-Net is capable of leveraging hierarchical features from multiple convolutional layers [30] and has been applied in the SR and denoising of computed tomographic images [31,32] and magnetic resonance images [33]. Lastly, LTE is a dominant-frequency estimator for images capable of characterizing textures in 2D Fourier space. Lee et al., showed that LTE achieved a more favorable performance than other deep learning-based SR models [19]. This approach is relatively new and has not been applied to medical imaging before. It emphasizes learning high-frequency details, such as the edges, in order to improve clinicians’ diagnostic confidence in detecting lesions and structures.

No previous study has compared the performance of these recent models for SR of panoramic radiographs. In the present study, while all of the selected models showed promising outcomes, LTE showed the highest MSE, PSNR, SSIM, and MOS, respectively. However, our findings show that LTE is more computationally expensive, which is a drawback.

In this study, we evaluated the correlation between clinician perception of image quality through MOS and objective measurements (MSE, PSNR, and SSIM). While all of them showed strong correlations, these were not all in the same direction. It has previously been reported that MOS and other metrics may not necessarily agree [16], by large as the objective metrics reflect different image properties. This is why a more comprehensive set of objective metrics has been suggested [9]. Moreover, a variety of factors, including emotion, professional background, and personal experience have been shown to affect the results of subjective evaluations [34], which is why we employed different examiners to mitigate this variance to some degree. Overall, the main messages of the different employed metrics remain similar across metrics in the present study, strengthening the case to employ both subjective and objective measures [9]. Moreover, the most inconsistency was observed in SRGAN output. Such discrepancies have previously been shown for SRGAN. It is generally determined that SR models trained with adversarial loss and content loss achieve lower PSNR than those trained with pixel loss, while significantly improving perceived quality [9].

The application of SR models for enhancing image quality has several advantages. SR allows zooming into radiographs with appropriate quality. Radiographic images with higher quality can facilitate diagnosis and better treatment planning in different clinical settings. Additionally, employing SR may decrease the radiation dose by eliminating the need for radiographic retakes and additional imaging due to poor resolution. Moreover, radiographic quality is often positively correlated with radiation dose [35]. Lastly, the combined or specific application of different SR approaches may allow targeted SR for particular tasks (e.g., caries detection, measurement of periodontal bone loss, and detection of periapical lesions), partially because many of these tasks also require assessment of different anatomic regions. Software manufacturers may want to explore the option of condition-specific SR.

The findings of this study showed that objective assessments by evaluation metrics and subjective analysis by expert opinion do not necessarily agree. This can be attributed to the small sample size or interference of image characteristics other than resolution in the viewers’ scoring. One of the main limitations of this study was the limited number of training images. Larger datasets may improve model performance and generalizability. For further research, the effects of SR models on the quality of cone beam computed tomography (CBCT) images should be explored. Since radiation dose is a major concern in three-dimensional images in dentistry, applying SR models to enhance the quality of low-dose and low-resolution CBCT scans is promising.

## 5. Conclusions

It is possible to improve the visual quality of images by applying SR methods via deep learning models. However, for enhancing the quality of panoramic radiographs, the LTE and SRCNN models were the models that showed the most desirable improvement in both subjective and objective measures of quality. Nevertheless, future research should evaluate whether these improvements lead to better diagnostic capabilities as a result of these models. Furthermore, future studies should take into account the possibility that different SR approaches may be appropriate for different conditions.

## Figures and Tables

**Table 1:** The definition of technical terms.

Term	Definition
Batch size	The number of image samples in each batch of the dataset used for training model
Early stopping	Assessing the performance of the model during training and stopping training at some criterion to reduce overfitting
Epoch	One training cycle
Hyperparameter	Non-learnable parameters that affect the training
Hyperparameter tuning	A strategy to find optimal hyperparameters
Learning rate	A hyperparameter that is used to adjust learning speed
Optimizer	A hyperparameter that is used to adjust learnable parameters
Overfitting	A model overfits when it begins to memorize training data instead of developing generalizable patterns

**Table 2:** Objective outcomes of super-resolution using different approaches on the test set. We provide the mean values for MSE, PSNR, and SSIM.

Approach	MSE	PSNR	SSIM
Bicubic	25.74	34.02	0.890
SRCNN	7.48 ± 0.30	39.57 ± 0.16	0.917 ± 0.004
SRGAN	22.31 ± 0.37	34.43 ± 0.32	0.901 ± 0.005
U-Net	8.55 ± 0.23	38.76 ± 0.28	0.917 ± 0.003
SwinIR	17.31 ± 0.21	36.53 ± 0.35	0.916 ± 0.001
LTE	7.42 ± 0.44	39.74 ± 0.17	0.919 ± 0.003

MSE, mean squared error; PSNR, peak signal-to-noise ratio; SSIM, structural similarity index.

**Table 3:** *p*-Values for the comparison of outcomes using different approaches.

Approach	MSE					PSNR					SSIM				
	SRCNN	SRGAN	U-Net	SwinIR	LTE	SRCNN	SRGAN	U-Net	SwinIR	LTE	SRCNN	SRGAN	U-Net	SwinIR	LTE
Bicubic	<0.001	<0.001	<0.001	<0.001	<0.001	<0.001	0.046	<0.001	<0.001	<0.001	<0.001	<0.001	<0.001	<0.001	<0.001
SRCNN	-	<0.001	<0.001	<0.001	0.801	-	<0.001	<0.001	<0.001	0.206	-	<0.001	1	0.588	0.742
SRGAN	-	-	<0.001	<0.001	<0.001	-	-	<0.001	<0.001	<0.001	-	-	<0.001	<0.001	<0.001
U-Net	-	-	-	<0.001	<0.001	-	-	-	<0.001	<0.001	-	-	-	0.959	0.584
SwinIR	-	-	-	-	<0.001	-	-	-	-	<0.001	-	-	-	-	0.068

MSE, mean squared error; PSNR, peak signal-to-noise ratio; SSIM, structural similarity index.

**Table 4:** The subjective outcome of super-resolution approaches, measured via MOS. The *p*-value indicates differences compared with LR images.

Approach	Rater 1	Rater 2	Rater 3	Rater 4	Total MOS (Mean ± sd)	*p* Value
SRCNN	2.87	3.81	3.27	3.84	3.45 ± 0.75	<0.001
SRGAN	3.03	3.39	3.19	3.45	3.27 ± 0.65	<0.001
U-Net	2.93	3.39	2.97	3.56	3.21 ± 0.86	0.002
SwinIR	2.94	3.23	2.81	3.31	3.07 ± 0.77	0.017
LTE	3.43	3.73	3.43	3.77	3.59 ± 0.54	<0.001
HR image	3.78	4.13	3.77	4.20	3.97 ± 0.53	<0.001
Bicubic	2.1	2.91	2.67	3.03	2.68 ± 0.77	-

MOS, mean opinion score; HR, high resolution; LR, low resolution.

## Data Availability

The data that support the findings of this study are available from the corresponding author upon reasonable request.
